# Improving Outcomes through Implementation of an Infant Spinal Anesthesia Program for Urologic Surgery Patients

**DOI:** 10.1097/pq9.0000000000000615

**Published:** 2023-05-22

**Authors:** Jessica A. Cronin, Brenda Satterthwaite, Giannina Robalino, Daniel Casella, Michael Hsieh, Md Sohel Rana, Alia Fink, Sophie Pestieau

**Affiliations:** From the * Division of Anesthesiology, Pain and Perioperative Medicine, Children’s National Hospital, Washington, D.C.; † Division of Urology, Children’s National Hospital, Washington, D.C.; ‡ The Joseph E. Robert Jr., Center for Surgical Care, Children’s National Hospital, Washington, D.C.; § Performance Improvement Department, Children’s National Hospital, Washington, D.C.

## Abstract

**Introduction:**

Spinal anesthesia has a long history as an effective and safe technique to avoid general anesthesia in infants undergoing surgery. However, spinal anesthesia was rarely used as the primary anesthetic in this population at our institution. This healthcare improvement initiative aimed to increase the percentage of successful spinal placements as the primary anesthetic in infants undergoing circumcision, open orchidopexy, or hernia repair from 11% to 50% by December 31, 2019, and sustain that rate for 6 months.

**Methods:**

An interdisciplinary team created a key driver diagram and implemented the following interventions: education of nurses, surgeons, and patient families; focused anesthesiologist training on the infant spinal procedure; premedication; availability of supplies; and surgical schedule optimization. The team collected data retrospectively by reviewing electronic medical records (Cerner, North Kansas City, Mo.). The primary outcome was the percentage of infants undergoing circumcision, open orchidopexy, or hernia repair who received a successful spinal as the primary anesthetic. The team tracked this measure and evaluated using a statistical process control chart.

**Results:**

Between August 1, 2018, and February 29, 2020, researchers identified 470 infants (235 preintervention and 235 postintervention) who underwent circumcision, open orchidopexy, or inguinal hernia repair. Following the interventions in this project, there was a statistically significant increase in successful spinal placement from 11% to 45% (*P* < 0.0001).

**Conclusion:**

This quality improvement project successfully increased the percentage of patients receiving spinal anesthesia for specific surgical procedures by increasing the number of patients who underwent successful spinal anesthesia placement.

## INTRODUCTION

### Problem Description

Adverse effects of general anesthesia (GA) on infants (children younger than 1 year) include respiratory complications, delayed postanesthesia care unit (PACU) discharge, and unplanned hospital admissions. In addition, while some reassuring studies have demonstrated the safety of anesthetic agents on the developing brain, the data are still inconclusive, particularly for patients with longer, more frequent anesthetic exposures.^1,2^ At the study institution, approximately 2,600 surgeries are performed yearly on infants. However, these surgeries are rarely performed under spinal anesthesia as the primary anesthetic.

### Available Knowledge

Spinal anesthesia has a long history as an effective, safe, and well-tolerated technique to avoid GA in young infants undergoing surgery, particularly in patients with multiple comorbidities.^3,4^ Compared with GA, a spinal anesthetic in this population decreases intraoperative blood pressure changes, airway manipulation, operating room (OR) time, and PACU length of stay.^5–8^ Furthermore, this neuraxial anesthesia technique avoids potential neurotoxicity secondary to GA.^9^

### Rationale

Multiple institutions have safely and effectively initiated infant spinal programs into everyday practice.^2,4,6^ Using these institutions’ experience regarding patient selection, medication dosing, and procedural approach, we developed a framework for our infant spinal program that met the needs of our hospital.

### Specific Aim

The primary aim of this quality improvement study was to identify and circumvent the barriers to using spinal anesthesia in infants undergoing circumcision, open orchidopexy, or inguinal hernia repair; increase the percentage who receive successful spinal anesthesia as the primary anesthetic from 11% to 50% by December 31, 2019; and sustain that rate for 6 months.

## METHODS

### Context

The study occurred at an urban, tertiary care pediatric hospital in Washington, DC, with an annual patient surgical volume of approximately 18,000 cases per year, with 300 circumcisions, orchidopexies, and inguinal hernia repairs performed on infants annually. In early 2018, anesthesiologists began performing infant spinal anesthesia placements as an alternative to GA.

### Intervention

**Fig. 1.**
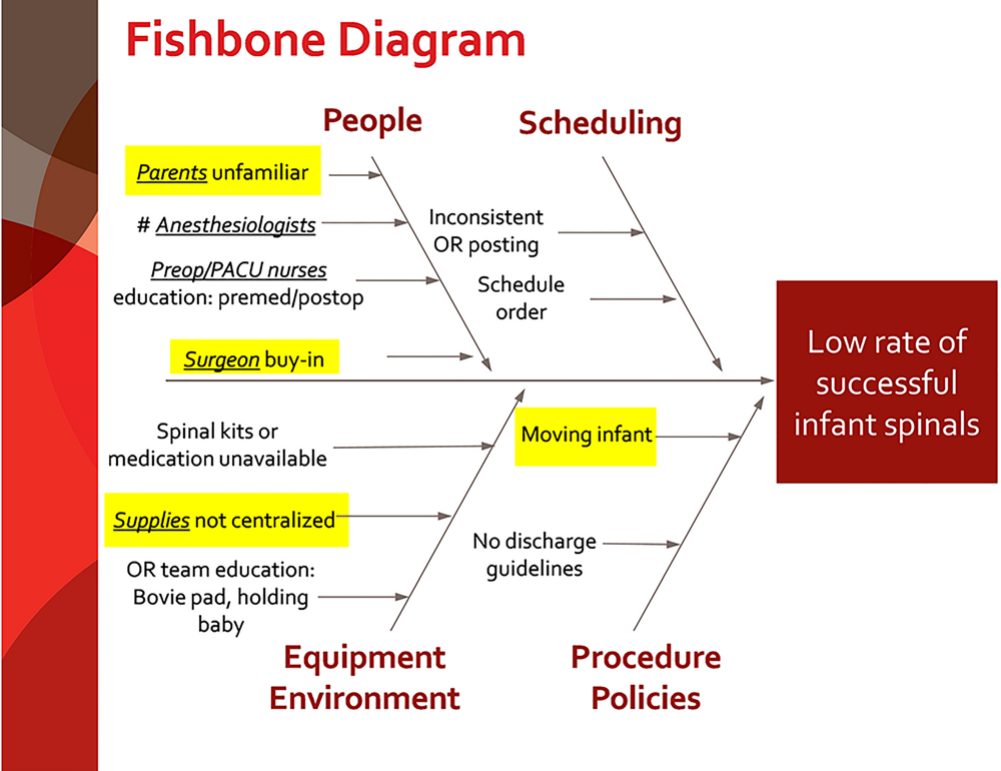
Fishbone diagram.

**Fig. 2.**
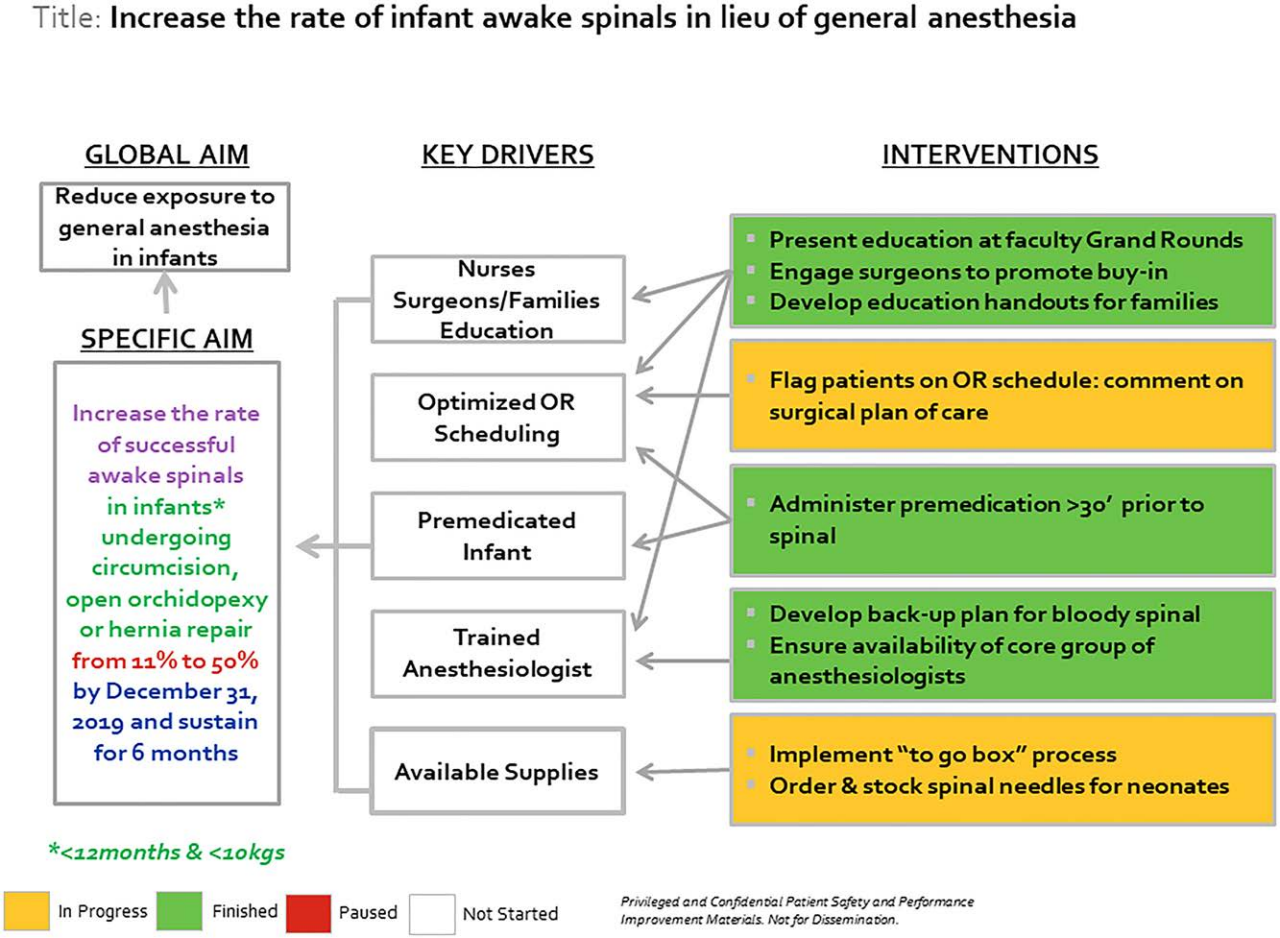
Key driver diagram.

A current state analysis was performed on data from August 1, 2018, to May 1, 2019, to determine the incidence of spinal anesthetics in this patient population. Following the current state analysis, our team of 5 anesthesiologists and 2 urologic surgeons created a specific aim to increase the rate of infant spinal placement to 50% by December 31, 2019, and sustain that rate for 6 months. Initially, the team developed a fishbone diagram to categorize causes for a low rate of successful infant spinals, including equipment and environment, procedures and policies, people, and scheduling (Fig. 1). Next, the team developed a key driver diagram based on issues identified in the fishbone diagram to achieve the specific aim (Fig. 2). Key drivers included educating nurses, surgeons, and families; anesthesiologist training; patient premedication; supply availability; and OR schedule optimization. Finally, the team devised the following countermeasures.

#### Education of Nurses, Surgeons, and Families

At the beginning of the project, 2 urologists encouraged families to consider infant spinals as an anesthesia option for their child’s surgery. Starting June 1, 2019, the team began offering educational lectures to perioperative nursing staff that focused on spinal anesthesia techniques, benefits, potential complications, and managing these infants in the preoperative and postoperative periods. In addition, the team engaged additional urology colleagues and reviewed with them the benefits of infant spinal anesthesia and which surgeries would qualify for spinal anesthesia. Patients’ families received a preoperative informational handout during their preoperative surgical visit that outlined a description of spinal anesthesia and its risk and benefits. (**See figure, Supplementary Digital Content 1,** http://links.lww.com/PQ9/A435.)

### Anesthesiologist Training

Starting June 1, 2019, we formed a core group of 5 pediatric anesthesiologists with expertise in regional anesthesia and, more specifically, infant spinal anesthesia. Before this date, anesthesiologists with variable degrees of experience placed spinal anesthetics. This group of 5, designated as the “infant spinal team,” developed a standardized pathway that included patient selection criteria, guidelines for spinal medication placement and dosing, and what to do when spinal alone does not achieve sufficient anesthesia. (**See figure, Supplementary Digital Content 2**, http://links.lww.com/PQ9/A436.) The patient selection criteria included: infants less than 1 year of age, weight less than 10 kg, absence of congenital cardiac disease and neurological disorders, absence of spinal cord malformations, and the scheduled surgery (inguinal hernia, circumcision, and orchidopexy). We used 0.75% hyperbaric bupivacaine for spinal medication dosing. For patients weighing less than 5 kg, 1 mg/kg was administered intrathecally, while patients between 5 and 10 kg received 0.5 mg/kg. Postspinal placement, an IV was placed in the lower extremity. Sweet-Ease (sucrose and purified water solution by Phillips Healthcare, Andover, Mass.), IV fentanyl, or IV dexmedetomidine could be given intraoperatively if the infant is not tolerating the procedure. Adjustments were made to the daily OR schedule so that at least 1 team member was available daily. The team also provided a grand round presentation to our division faculty to review the benefits, risks, and complications of spinal anesthesia; and the infant spinal program, including patient selection criteria, dosing guidelines, and a step-by-step guide for infant spinal anesthesia placement. An infant spinal anesthesia guide was posted online for easy access to a summary of this information. (**See figure, Supplementary Digital Content 2**, http://links.lww.com/PQ9/A436.)

#### Patient Premedication

Before this quality improvement project, the team identified increased difficulty placing spinal anesthesia in moving infants, especially those older than 6 months. To mitigate this and increase the likelihood of successful spinal anesthesia placement, starting on June 1, 2019, anesthesiologists administered intranasal dexmedetomidine 2−4 mcg/kg 30 minutes before spinal anesthetic placement. Following the administration of intranasal dexmedetomidine, patients were placed on continuous pulse oximetry, and the preoperative nurse was made aware of the premedication. We removed potential spinal candidates from first starts to accommodate earlier admission into the preoperative area, thus providing ample time to administer the premedication. We selected dexmedetomidine for premedication in these patients due to evidence showing the absence of adverse neurocognitive effects in infants associated with this medication.

#### Supply Availability

Before the project, we would gather supplies from 4 separate locations within the OR for each infant spinal anesthesia placement. Therefore, starting June 1, 2019, the team created a “to go” box that contained all the supplies required for infant spinal placement in the anesthesia central supply room. These boxes included a 1.5-inch 22-gauge spinal needle, a 1-ml syringe, a flush syringe, a ChloraPrep (patient preoperative skin preparation; Bectin, Dickinson, and Co., Franklin Lakes, N.J.) stick, a pacifier, and Sweet-Ease. Chloroprep was selected as an antiseptic based on reports supporting chlorhexidine-based solutions as the antiseptic of choice for regional anesthetics, including spinal anesthesia. Chlorhexidine skin antisepsis has shown significant advantages over povidone-iodine in terms of onset, efficacy, and potency without increasing the risk of neurologic complications.^10^

The OR technicians were responsible for assembling these “to go” boxes. This intervention ensured appropriate supplies would be in the OR promptly while minimizing delays.

#### OR Schedule Optimization

To help identify infants who were possible candidates for spinal anesthesia, the team asked surgical colleagues to indicate “spinal anesthesia” on the surgical posting. This notification informed all perioperative staff of potential spinal anesthesia candidates. In addition, before this quality improvement project, these patients were often scheduled to be the first case of the day to minimize fasting times. However, due to the registration process for first-case patients at our institution, it was often difficult to administer the premedication early enough to be effective during spinal anesthesia placement. Starting October 1, 2019, infant spinal anesthesia candidates were scheduled as a second or third case of the day. Families were given preoperative instructions to minimize fasting times as outlined by the American Society for Anesthesiologists.^11^

### Measures

The primary aim of this study was to increase the percentage of infants undergoing circumcision, open orchidopexy, or inguinal hernia under a spinal anesthetic versus a general anesthetic. We calculated this outcome before interventions to identify the baseline and then on an ongoing basis in a control chart after June 1, 2019. The data collection for spinal anesthesia cases (both successful and failed attempts) was by retrospective chart review of electronic anesthesia records, nursing records, and medication administration records. Electronic medical record data were extracted to query all patients who underwent circumcision, open orchidopexy, or inguinal hernia repair.

Our secondary measure was the percentage of successful spinal anesthesia placements compared with failed spinal anesthesia placements. Failures included all patients who had to undergo GA at any point during the procedure because of unsuccessful or incomplete spinal block.

The process measure was the percentage of patients who received dexmedetomidine premedication before spinal placement. In addition, as a balancing measure, the time between the patient’s arrival in the OR and spinal anesthesia placement and the start of the surgery was collected. No other compliance data or process metrics were collected.

### Analysis

We plotted process and outcome measures on run and control charts, and special versus common cause variations were differentiated using established rules.^12^ Demographic and spinal anesthesia-related data were compared between the preintervention and postintervention groups using unpaired *t*-test for continuous data and Chi-square test or Fisher exact test (if any of the expected cell sizes were <5) for categorical data. Unless otherwise stated, all statistical tests were 2-sided and performed at the 5% significance level. We used Stata 15.1 software (StataCorp, College Station, Tex.) for all statistical analyses.

### Ethical Considerations

As a quality improvement project and not human subject research, the project did not require review and approval by the institutional review board.

## RESULTS

Several demographic differences existed between the preintervention and postintervention groups of infants with successful spinal placement (Table 1). The preintervention infants weighed less (6.6 ± 1.7 kg versus 8.0 ± 1.9 kg, *P* < 0.0001) and were younger (18.4 ± 9.3 weeks versus 28.4 ± 11.4 weeks, *P* < 0.001) than postintervention infants. There was no difference in the history of prematurity between the 2 groups.

**Table 1.** Demographic Data for Patients Who Underwent Successful Spinal Anesthesia Placements

Variables	Preintervention	Postintervention	*P*
n	27	106	
Weight (kg)	6.6 (1.7)	8.0 (1.9)	**<0.001**
Age (wk)	18.4 (9.3)	28.4 (11.4)	**<0.001**
% History of prematurity	3 (11.1)	12 (11.3)	0.98
In room to spinal placement (min)	9.7 (3.6)	8.9 (4.1)	0.35
In room to surgery start (min)	22.6 (5.7)	21.8 (7.6)	0.65
Total qualifying procedures (n)	235	235	
% Successful spinals of total qualifying procedures	27 (11.5)	106 (45.1)	**<0.001**
Total failed spinals (n)	10	13	
% Spinals attempted of total qualifying procedures	37/235 (15.7)	119/235 (50.6)	**<0.001**
% Successful spinals of total attempted spinals	27/37 (73.0)	106/119 (89.1)	**0.016**

When not otherwise specified, data reflect mean (standard deviation)

Boldface value indicates *P*<0.05.s

**Fig. 3.**
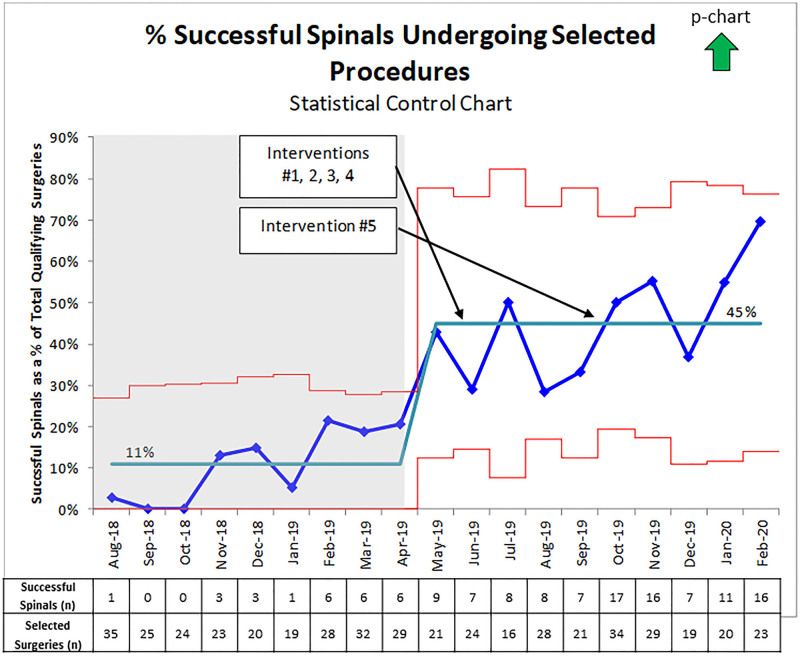
Percentage of successful spinal anesthesia placements in infants undergoing circumcision, open orchidopexy, or inguinal hernia repair. Note: Intervention 1 was the education of nurses, surgeons, and families. Intervention 2 was anesthesiologist training. Intervention 3 incorporated patient premedication as part of the standard of care. Intervention 4 was increasing supply availability. Finally, intervention 5 was OR schedule optimization—additional information regarding these interventions in Methods.

Before this quality improvement project, 11% of the circumcisions, open orchidopexies, and inguinal hernia repairs were performed in infants using spinal anesthesia as the primary anesthetic. Between August 1, 2018, and February 29, 2020, 470 infants underwent circumcision, open orchidopexy, or inguinal hernia repair (235 preintervention and 235 postintervention). One hundred thirty-two of these infants underwent a successful spinal anesthetic (26 preintervention and 106 postintervention). Following the interventions, there was an increase in successful spinal placements to 45% from 11% (*P* < 0.0001), with a statistically significant shift in the primary outcome due to achieving 8 data points above the baseline (Fig. 3).

**Fig. 4.**
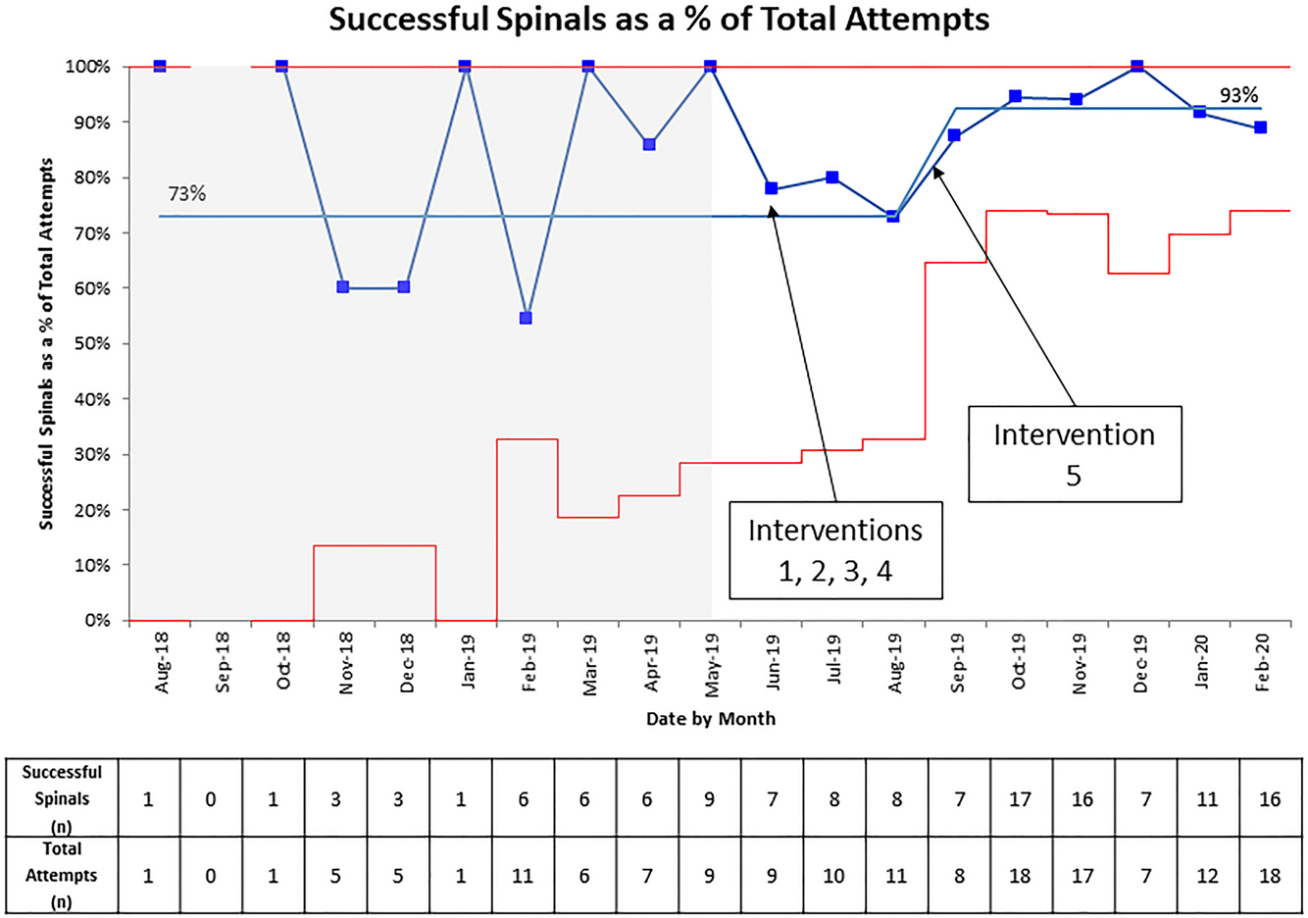
Percentage of successful spinal anesthesia placements as a percent of total attempts.Note: Intervention 1 was the education of nurses, surgeons, and families. Intervention 2 was anesthesiologist training. Intervention 3 incorporated patient premedication as part of the standard of care. Intervention 4 was increasing supply availability. Finally, intervention 5 was OR schedule optimization—additional information regarding these interventions in Methods.

**Fig. 5.**
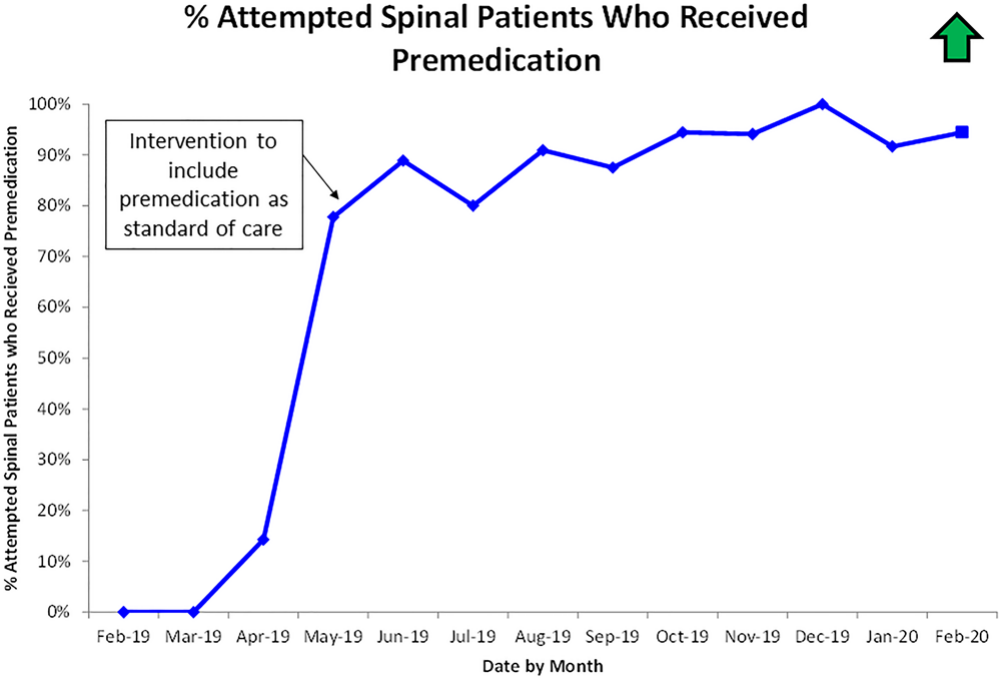
Process measure—the percentage of patients who attempted spinal anesthesia placement who received premedication. Note: additional information regarding these interventions in Methods.

Before interventions, the average success rate of infant spinal anesthesia was 73% (Table 1). Following the interventions, the success rate was 89% (Table 1). The statistical control chart in Figure 4 shows the corresponding centerline shift following interventions. Analysis of the process measure showed the percentage of attempted spinal anesthesia patients who received dexmedetomidine premedication increased from 0% in March 2019 to 91% between June 2019 and February 2020 (Fig. 5).

As part of our balancing measure, we looked at the time between the patient’s arrival in the OR and spinal anesthesia placement and between arrival in the OR and the start of surgery. We found no differences in duration from patient in-room-time to spinal placement between the preintervention group (9.7 min ± 3.6) and the postintervention group (8.9 min ± 4.1). There was also no difference between the groups in duration of arrival into the OR to the start of surgery (22.6 min ± 5.7 preintervention versus 21.8 ± 7.6 min postintervention) (Table 1). These times were not significantly different than for the patients who underwent GA (26.8 min ± 15.88).

There were no complications, including unplanned admissions, bleeding, or adverse reactions, due to spinal placement or attempts throughout the project.

## DISCUSSION

### Summary

As a result of this quality improvement project, the percentage of infants who successfully underwent spinal anesthesia for circumcision, open orchidopexy, or inguinal hernia repair increased from 11% to 45% at our institution. While the specific aim was not achieved, there was a significant increase in the number of urological procedures successfully performed under spinal anesthesia in children less than 1 year of age. In addition, the success rate among the infants who underwent a spinal anesthesia placement increased from 73% preintervention to 89% postintervention (Table 1). Our process measure of dexmedetomidine administration before spinal anesthesia placement increased from 0% to 91% during the study. The balancing measure for the study demonstrated no difference in OR times in patients who had undergone our interventions, including dexmedetomidine administration.

### Interpretation

There was a significant difference in demographic data between patients who underwent successful spinal placement preintervention versus postintervention. Patients in the preintervention group were younger and weighed less than that in postintervention (Table 1). Before this project, spinal anesthesia at this institution was primarily performed on infants at high risk for undergoing GA. This initial population consisted predominantly of neonates and very young infants in the neonatal intensive care unit—the smallest, sickest, and youngest surgical patients. Following education, more surgeons were interested in performing their procedures under spinal anesthesia. This change led to an increase in the number of healthy, outpatient, and older infants who received spinal anesthesia, accounting for the shift in demographics preintervention compared with postintervention.

At the beginning of June 2019, we implemented 5 key drivers, including education of nurses, surgeons, patient families, and anesthesiologists; infant premedication; and improvement in supplies availability. With these key drivers in place, the team noted our first sustained increase in successful infant spinal anesthesia placements as a percentage of total qualifying procedures to 30%−50% (Fig. 3). In October 2019, OR schedule optimization increased the primary outcome to greater than 40%, although this increase has not met the requirements for another baseline shift. There are a few possible explanations for why we could not reach our goal of 50%, including variability in the experience of physicians placing spinals since we included fellows in our spinal anesthesia placement. Additionally, parental consent for spinal may have limited the pool of candidates eligible for spinal placement.

Before this quality improvement project, the success rate for infant spinal anesthesia placement, the secondary measure, was 73%. Success rates at other institutions with established infant spinal anesthesia programs range between 80% and 95% depending on the age of the infant and provider experience.^6,13,14^ This program’s success increased between June 2019 and December 2019, with a transient decrease in August 2019. This decrease may have resulted from starting a new pediatric anesthesiology fellow class in August with minimal or no infant spinal anesthesia experience. While it is difficult to distinguish which intervention was most effective in improving this metric, given the timing of the interventions, the team attributed success to the training and availability of a core group of anesthesiologists and consistent dexmedetomidine premedication to improve spinal anesthesia placement conditions. At the end of the quality improvement project, this institution’s performance on this metric was consistent with other leading institutions.

Balancing measures that were assessed during the study were the time between the patient’s arrival in the OR and spinal anesthesia placement and the time between arrival in the OR and the start of surgery. OR throughput would suffer if times were to increase during this quality improvement project. The in-room-to-spinal-placement time and in-room-to-surgery-start time did not vary significantly between the preintervention and postintervention groups (Table 1) or the patients who underwent GA. The absence of difference between these times demonstrates that the multiple interventions throughout the project did not affect OR time efficiency. This lack of negative impact on throughput may also have encouraged surgeons initially concerned to consider this approach.

### Limitations

There are several limitations to the project. A specially trained anesthesiologist group performed the spinal anesthesia placements, which may not be available at every institution. Also, this study examined patients undergoing urological procedures; therefore, the results may not be generalizable to other procedures performed under spinal anesthesia. The patients included in this study were less than 1 year of age. Our improvement may not apply to spinal anesthesia outside of this age range. Also, none of the study patients had any cardiac disease; therefore, study results may not apply to that specific population of patients.

## CONCLUSIONS

Our project, guided by the key drivers, aimed to increase the number of urological procedures successfully performed under spinal anesthesia in children less than 1 year of age. The interventions increased the number of patients who underwent spinal anesthesia placement. They also increased the success rate of spinal anesthesia placements. Further quality improvement efforts may increase the number of infants considered for infant spinal anesthesia as primary anesthesia for their procedure, beyond circumcision, open orchidopexy, and inguinal hernia repair.

## DISCLOSURE

The authors have no financial interest to declare in relation to the content of this article.

## Supplementary Material


