# Supplementary material for: Improving Outcomes through Implementation of an Infant Spinal Anesthesia Program for Urologic Surgery Patients
Source: Pediatr Qual Saf. 2023 May 22;8(3):e615. doi: 10.1097/pq9.0000000000000615 (PMC10990379; doi:10.1097/pq9.0000000000000615)
Supplement: Supplementary file 1 [file pqs-8-e615-s001.pdf]

Children's National

Patient Education- Spinal Anesthesia

What is spinal anesthesia?

- Spinal anesthesia involves a single injection of local anesthetic (numbing medication) around the spinal cord. This local anesthetic numbs the nerves that supply pain sensation and motor function to the lower abdomen and legs.
- The numbing medication lasts for 1-2 hours and allows surgical procedures to be performed without any sensation of pain or feeling by the child.
- After numbing the skin of the back, an experienced anesthesiologist (special trained doctor) will place the spinal anesthetic. To reduce medication exposure, the spinal is performed while your child is awake. Your child's lower abdomen, legs and feet will quickly be numbed, at which time an IV will be placed in your child's foot. The IV allows the anesthesiologist to give medications and fluids.
- Your child may be given a pacifier and sugar water for comfort while they undergo the spinal anesthetic and their surgical procedure. An anesthesiologist will be caring for your child during the entire surgery to monitor your child's vital signs and assess their level of comfort.

What are the benefits of spinal anesthesia?

- Spinal anesthesia allows your child to have surgery without exposure to general anesthesia. General anesthesia would only be administered if the spinal does not fully numb your child's lower abdomen and legs or if there are problems with placement. General anesthesia requires anesthetic gases and other medications. It will also require placement of a breathing tube during the surgery. Multiple studies have shown that infants who undergo spinal anesthesia instead of general anesthesia are at decreased risk respiratory events, airway complications, and heart rate changes during the surgery.
- Recent studies have shown that brief exposures to general or spinal anesthesia in otherwise healthy infants does not affect brain development. Spinal anesthesia provides a way to perform your child's surgery without exposing them to these additional anesthetic medications.

What are the risks involved with spinal anesthesia?

- Spinal anesthesia has been performed in infants since the 1980s, with multiple reports on its safety.
- Risks of spinal anesthesia include the possibility of bleeding (at the injection site), heart rate and blood pressure changes, medication side effects, inability to place the spinal anesthetic requiring general anesthesia, and damage to nerves and tissues near the injection site.

What should I expect the day of surgery?

- Your child's anesthesiologist will meet with you before the procedure to discuss the anesthetic plan. If your anesthesiologist thinks a spinal anesthetic is the best option for your child, then your child may be given some relaxing medication (through the nose) to facilitate placing the spinal.
- When it is time for the procedure, your child will be taken to the operating room where the spinal will be performed.
- Upon completion of the surgery, your child will be transported to the recovery room. If your child is under 6 months, expect to stay in the recovery room for at least 2 hours for monitoring. Premature infants and infants less than 2 months of age may be required to stay overnight for additional monitoring per hospital policy.
