# Supplementary material for: Improving Outcomes through Implementation of an Infant Spinal Anesthesia Program for Urologic Surgery Patients
Source: Pediatr Qual Saf. 2023 May 22;8(3):e615. doi: 10.1097/pq9.0000000000000615 (PMC10990379; doi:10.1097/pq9.0000000000000615)
Supplement: Supplementary file 2 [file pqs-8-e615-s002.pdf]

Infant Spinal Anesthesia Guide

Selection Criteria
1. Age < 18 months
2. Surgery time < 60 minutes
3. Surgery below umbilicus
    a. Inguinal Hernia Repair (Open only, laparoscopic contraindicated)
    b. Orchidopexy
    c. Circumcision
    d. Multiple procedures may qualify, discuss with surgeon as appropriate

Supplies
1. Spinal Tray (contains lidocaine 1%, hyperbaric bupivacaine 0.75%)
2. Spinal Anesthesia Kit (contains pacifier, sucrose, TB syringe, clear chloraprep, 22G 1.5-inch spinal needle x 2, NaCl flush syringe)
    a. Can be found next to Spinal Trays in anesthesia stock room
    b. Use 25G 2-inch spinal needle for neonates (found separately in main anesthesia stock room)
3. Intranasal dexmedetomidine: best administered via atomizer
4. Bovie pad out of package (ready to place on infant's back as soon as spinal is completed)

Spinal Anesthesia Dosage for Infants (<12 months)

Medications	Suggested Dose[1] (mg/kg)	Block Duration (mean)	Comments
Bupivacaine 0.75% Hyperbaric	< 5kg = 1mg/kg<br>5-10kg = 0.5mg/kg<br>10-15kg = 0.4mg/kg	60-90 minutes (75)	Usual max dose:<br>< 12 months = 7.5mg
Bupivacaine 0.5% Isobaric		30-180 min (80)	
Ropivacaine 0.5%		35 – 240 min (90)	

Epinephrine	2-3 mcg/kg or epi wash	Increase block duration 30-40%

Premedication: 3-4 mcg/kg intranasal dexmedetomidine **20-30 minutes prior to surgery start**

Positioning: Sitting or lateral
- Bring OR table up to appropriate level, easier to stay midline
- For sitting position place thumb on humerus & digit 5 on femur
    - 2 blankets to lay on infant's lap (if sitting position)
    - Doughnut for sitting infants

Spinal Placement:
- Use 1 cc syringe to draw up and administer bupivacaine
- Stay midline (lateral = blood)

Post-Spinal Placement:
- All monitors on lower extremities (IV, BP, pulse ox)
- Place bovie pad on back (**roll the patient, do NOT lift legs → high spinal**)

During the Procedure:
- Towel under neck to support head
- Arms up and free (thumb suckers)
- Wrap drape around elbows with drape clips

Inadequate Block: consider caudal + sedation
- Caudal: 0.25% bupivacaine (central pyxis) or 0.2% ropivacaine (less potent than bupivacaine)
    - Dose: 1mL/kg
- IV dexmedetomidine 0.5-1mcg/kg PRN
- Fentanyl 1-2mcg/kg PRN

End of Surgery:
- Field block by surgeon or caudal 0.2% ropivacaine: **dose should be 0.5-0.75 mL/kg**
- **<u>Keep infant flat</u>** (bovie pad removal, diaper placement)

[1] Adapted from:
ASRA-ESRA 2018 Recommendations. *Reg Anesth Pain Med* 2018;43: 211–216.
Vermont Database
Nationwide Childrens Experience
